# The ratio of AGE to sRAGE independently associated with albuminuria in hypertensive patients

**DOI:** 10.1186/s12902-018-0306-7

**Published:** 2018-11-13

**Authors:** Kuang-Hsing Chiang, Jaw-Wen Chen, Shao-Sung Huang, Hsin-Bang Leu, Shing-Jong Lin, Po-Hsun Huang

**Affiliations:** 1 Division of Cardiology and Cardiovascular Research Center, Department of Internal Medicine, Taipei Medical University Hospital, Taipei, Taiwan; 2 Graduate Institute of Biomedical Electronics and Bioinformatics, National Taiwan University, Taipei, Taiwan; 3 Division of Cardiology, Department of Internal Medicine, Taipei Veterans General Hospital, Taipei, Taiwan; 4 Department of Medical Research and Education, Taipei Veterans General Hospital, Taipei, Taiwan; 5 Cardiovascular Research Center, National Yang-Ming University, Taipei, Taiwan; 6 Institute of Pharmacology, National Yang-Ming University, Taipei, Taiwan; 7 Healthcare and Service Center, Taipei Veterans General Hospital, Taipei, Taiwan; 8 Institute of Clinical Medicine, National Yang-Ming University, Taipei, Taiwan; 9 Department of Internal Medicine, School of Medicine, College of Medicine, Taipei Medical University, Taipei, Taiwan

**Keywords:** Advanced glycosylation end products, Advanced glycosylation end product-specific receptor, Hypertension, Albuminuria

## Abstract

**Background:**

Soluble receptor for advanced glycation end-products (sRAGE) and advanced glycation end-products (AGE) have been associated with risks of cardiovascular disease. Because sRAGE is regarded as a scavenger to AGE, we hypothesized that the ratio of AGE to sRAGE (AGE/sRAGE) is associated with albuminuria in hypertensive patients.

**Methods:**

In this cross-sectional study, a total of 104 patients with essential hypertension were recruited. Hypertension was defined as a systolic blood pressure ≥ 140 mmHg, a diastolic blood pressure ≥ 90 mmHg, or use of antihypertensive treatment. Albuminuria was defined as albumin excretion rate ≧ 20 μg/min. Multivariate logistic regression analyses were performed to evaluate the association between AGE/sRAGE and albuminuria.

**Results:**

Among the 104 patients, 30 (28.8%) patients had albuminuria and 74 (71.2%) patients did not. Patients with albuminuria had higher AGE (2.15 vs. 1.71 μg/mL), lower sRAGE (424.5 vs. 492.5 pg/ml) and higher AGE/sRAGE (3.79 vs. 3.29 μg/pg) than those without albuminuria. Multivariate logistic regression model revealed that AGE/sRAGE (OR = 1.131, 95% CI = 1.001–1.278, *P* = 0.048) was independently associated with albuminuria. There was no significant relationship between AGE and sRAGE alone with albuminuria.

**Conclusion:**

This study suggests that the ratio of AGE to sRAGE may be a surrogate biomarker for microvascular injury. Further prospective studies of the prognostic value of the ratio in relation to microvasular injury are needed.

## Background

In hypertensive patients, the association between albuminuria and several cardiovascular risk factors has been widely demonstrated [1]. The existence of albuminuria is associated with signs of subclinical end-organ damage [2, 3] and is a strong indicator of microvascular damage in patients with essential hypertension [4]. Additionally, microalbuminuria is associated with an increased risk of all-cause mortality and cardiovascular mortality and is an early surrogate marker for cardiovascular events in hypertensive patients [5, 6]. Reduction of albuminuria was also associated with cardiovascular protection [7]. Combing conventional Framingham score for cardiovascular risks with albuminuria may exert higher efficiency to determine a primary prevention strategy [8].

Advanced glycation end-products (AGE) is a group of modified proteins or lipids which become glycated and oxidized after exposure to sugars. The interaction of AGE and receptor for advanced glycation end-products (RAGE) on the membrane of endothelial cells can activate intracellular signal cascade, elicit reactive oxygen species production, activate nuclear factor-κB, and increase gene expression and release of inflammatory cytokines, resulting in the progression of atherosclerosis [9]. Soluble RAGE (sRAGE), either cleaved proteolitically from the membranous receptor via matrix metalloproteinases or secreted endogenously, can bind with AGE as a decoy receptor and therefore may play a protective role by avoiding the AGE-RAGE interaction [10]. Animal models have showed that inhibiting AGE-RAGE axis by sRAGE could attenuate the development and progression of cerebrovascular and cardiovascular disease [11, 12].

Both AGE and sRAGE play a critical role in the process of atherosclerosis; however, a number of studies revealed controversial results for either AGE or sRAGE alone. Fujisawa et al. reported that sRAGE was positively associated with the risk of cardiovascular disease in type 2 diabetes patients [13]. But Falcone et al. reported an inversely association of sRAGE with the presence of coronary artery disease in non-diabetes patients [14]. Similar controversy was also noted for AGE. The association of AGE with cardiovascular events was significant in some situations [15, 16], but insignificant in others [17]. As commented by Prasad et al., a potential solution to the above mentioned controversy is to consider the AGE and sRAGE simultaneously because AGE-RAGE axis involves AGEs, cellular receptor RAGE, sRAGE, and endogenous secretory RAGE. In humans, cellular receptor RAGE cannot be measured and the serum concentration of sRAGE are five times higher than endogenous secretory RAGE in healthy subjects. Therefore, the ratio of AGE to sRAGE (AGE/sRAGE) might be the most appropriate biomarker for AGE-RAGE axis [18].

To date, numerous studies have investigated the association of AGE/sRAGE with various conditions, for example, adiponectin [19], log trimethylamine-N-oxide [20], vascular function [21] end-stage renal disease [22], and idiopathic pulmonary fibrosis [23]. However, there is a lack of studies on the role of AGE/sRAGE in early-stage of atherosclerosis. Because current evidence implied that identifying novel biomarkers which is associated with albuminuria is possibly a way to find a surrogate for early evaluation of future cardiovascular risk, this study aimed to test the hypothesis that increased AGE/sRAGE may associate with the existence of albuminuria in patients with essential hypertension.

## Methods

### Study subjects

In this cross-sectional study, a total of 104 patients with essential hypertension were recruited from the outpatient clinic at Taipei Veterans General Hospital. Hypertension was defined as a systolic blood pressure (SBP) ≥ 140 mmHg, a diastolic blood pressure (DBP) ≥90 mmHg, or use of antihypertensive treatment. None of the patients had a history or clinical evidence of diabetic nephropathy, chronic nephritis, renal failure, liver cirrhosis, inflammatory disease, or hematological disease. Patients with diabetes or taking anti-diabetes drugs were excluded. Secondary hypertension was also excluded by appropriate investigations, including measurement of plasma renin activity and aldosterone, Doppler studies of the renal arteries, renal scintigraphy or renal angiography. Medical history, including cardiovascular risk factors, previous and present cardiovascular events, and current drug treatment, was obtained during a personal interview and from medical records.

All patients gave written informed consent, and the study was approved by the Institute of Review Board in Taipei Veterans General Hospital (VGHIRB No: 96–12-42A).The protocols of this study were consistent with ethical guidelines provided in the 1975 Helsinki Declaration.

### Data collection

For the study, a detailed review of each patient’s chart and an interview was conducted to collect information on the patient’s symptoms, medications, coronary risk factors, smoking status, family history of coronary artery disease, and other systemic diseases. All participants underwent a standard clinical examination. The Framingham risk score was determined for each participant to estimate the 10-year incidence of cardiovascular events (angina, myocardial infarction, or death). This score was based on age, sex, total cholesterol, high density lipoproteins (HDL), diabetes, history of smoking, left ventricular hypertrophy, and SBP. Hyperlipidemia was defined as total cholesterol concentration of 200 mg/dl. The body mass index (BMI) was calculated by dividing the weight of the patient in kilograms by the square of the height in meters. Concentration of fasting blood glucose, uric acid, blood urea nitrogen, creatinine, triglycerides, total cholesterol, HDL cholesterol and low-density lipoprotein (LDL) cholesterol, serum total bilirubin, aspartate aminotransferase (AST), and alanine aminotransferase in blood samples drawn after a 12-h fasting were measured using a Hitachi 7600 autoanalyzer (Hitachi Ltd., Tokyo, Japan).

### Laboratory investigations

After a 12-h overnight fasting, blood samples were also collected to measure sRAGE, AGE, and high-sensitive C-reactive protein (hsCRP) concentrations. The blood samples were centrifuged at 3000 rpm for 10 min immediately after collection to obtain plasma and the plasma samples were kept frozen at − 70 °C until analysis. Concentration of sRAGE was determined using a commercially available enzyme-linked immunosorbent assay (ELISA) kit (Quantikine, R&D systems, Minneapolis, Minnesota, USA). Another commercial enzyme immunoassay (ELISA) kit (OxiSelect, Cell Biolabs, San Diego, California, USA) was used to detect a variety of AGE structures, including N-epsilon-(Carboxymethyl) lysine and pentosidine but not N-epsilon-(Carboxyethyl) lysine or methylglyoxal. Determination of hsCRP concentration was performed using a latex-enhanced immunophelometric assay (Dade Behring, Marburg, Germany) [19]. All procedures were carried out according to the instructions of the manufacturers and all measurements were performed by technicians who were blinded to all clinical data. Each standard and plasma sample was analyzed twice and the mean value was used in all subsequent analyses. The intra-assay and interassay variation coefficients were less than 10%.

Estimated creatinine clearance rate (eCCr) was calculated by the Cockcroft–Gault formula [20]. Morning urine samples were obtained for the creatinine to albumin ratio to determine the albumin excretion rate. Normoalbuminuria was defined as albumin excretion rate of < 20 μg/min, microalbuminuria was defined as albumin excretion rate of 20–200 μg/min, and macroalbuminuria was defined as albumin excretion rate > 200 μg/min.

### Statistical analysis

This study aimed to analyze the association of AGE/sRAGE with the presence of albuminuria in hypertensive patients. Patients with microalbuminuria or macroalbuminuria were grouped as ‘with albuminuria’ group and patients with normoalbuminuria as ‘without albuminuria’ group. Continuous data are presented as mean with standard deviation, and categorical data presented as count with percentage. The differences between patients with and without albuminuria for continuous and categorical data were tested using Mann-Whitney U test and Chi-square test, respectively. Multivariate logistic regression analyses with backward stepwise method were performed to evaluate the association between AGE/sRAGE and albuminuria with a *P*-value of less than 0.1 in the univariable analysis. A two-tailed P-value of < 0.05 indicated statistical significance. All statistical analyses were performed using IBM SPSS Statistics for Windows, Version 19.0 (Armonk, NY, USA).

## Results

### Sample characteristics

Among the 104 patients, 30 (28.8%) patients had microalbuminuria or macroalbuminuria, and 74 (71.2%) patients had normoalbuminuria. Demographic and clinical characteristics of the 104 hypertensive patients were provided in Table 1. Patients with albuminuria were older (58 vs. 56 years, *P* < 0.001), had significant higher SBP (150 mmHg vs. 137 mmHg, *P* = 0.002), higher DBP (83 vs. 78 mmHg, *P* = 0.039), higher pulse pressure (65 mmHg vs. 58 mmHg, *P* = 0.047), and more number of drugs (*P* = 0.027). Regarding the laboratory data, patients with albuminuria had significant higher AST (8 U/L vs. 6 U/L, *P* = 0.008). Patients with albuminuria had higher AGE (2.15 μg/mL vs. 1.71 μg/mL), lower sRAGE (424.5 pg/ml vs. 492.5 pg/ml) and higher AGE/sRAGE (3.79 μg/pg vs. 3.29 μg/pg) than those without albuminuria (Table 1).

**Table 1:** Demographic and clinical characteristics of study population

		Without albuminuria (*n* = 74)	With albuminuria (*n* = 30)	*P*
Age (year)		56 (51–66)	58 (46–71)	< 0.001
Gender^a^	Male	48 (64.9)	19 (63.3)	0.882
	Female	26 (35.1)	11 (36.7)	
Smoking^a^	Not smoker	45 (60.8)	19 (63.3)	0.839
	Past smoker	16 (21.6)	5 (16.7)	
	Current smoker	13 (17.6)	6 (20.0)	
Body mass index (kg/m^2^)		25.4 (23.9–27.3)	25.2 (23.7–27.0)	0.925
Heart rate (/min)		76 (68–86)	75 (72–84)	0.854
Systolic blood pressure (mmHg)		137 (126–147)	150 (132–158)	0.002
Diastolic blood pressure (mmHg)		78 (73–88)	83 (77–92)	0.039
Pulse pressure (mmHg)		58 (51–66)	65 (52–71)	0.047
Impaired fasting glucose^a^		36 (48.6)	16 (53.3)	0.829
Current medication				
ARB^a^		46 (62.2)	21 (70.0)	0.449
ACEI^a^		14 (18.9)	5 (16.7)	0.788
Calcium-channel blocker^a^		46 (62.2)	24 (80.0)	0.079
Diuretics^a^		17 (23.0)	8 (26.7)	0.690
Beta-blocker^a^		13 (17.6)	10 (33.3)	0.079
Statin^a^		9 (12.2)	3 (10.0)	1.000
Number of drugs		2 (1–3)	2 (2–3)	0.027
Laboratory				
Total cholesterol (mg/dl)		191.5 (170.0–214.0)	195.5 (182.0–214.0)	0.487
Triglycerides (mg/dL)		119.0 (76.0–184.0)	129.5 (102.0–197.0)	0.262
HDL (mg/dL)		42 (37–50)	43.5 (39–51)	0.586
LDL (mg/dL)		118.0 (95.6–140.0)	118.6 (101.0–133.4)	0.933
Fasting glucose (mg/dL)		99 (94–105)	100 (93–108)	0.950
ALT (U/L)		10 (8–13)	8 (7–11)	0.386
AST (U/L)		6 (5–9.5)	8 (7–11)	0.008
Uric acid (mg/dL)		6.05 (5.2–6.8)	6.5 (5.4–7.3)	0.264
eCCr (mL/min)		88.2 (72.9–104.6)	70.4 (55.0–103.0)	0.061
hs-CRP (mg/dL)		0.20 (0.16–0.36)	0.27 (0.16–0.55)	0.246
Adiponectin (μg/mL)		17.8 (11.0–27.6)	15.4 (11.2–25.1)	0.840
ADMA (μmol /L)		0.36 (0.33–0.40)	0.36 (0.32–0.41)	0.970
NT-proBNP (pg/mL)		84.1 (54.6–102.0)	93.7 (71.5–124.0)	0.077
AGE (μg/mL)		1.71 (0.82–2.45)	2.15 (0.87–2.65.00)	0.394
sRAGE (pg/ml)		492.5 (375.0–682.0)	424.5 (310.0–583.0)	0.200
AGE / sRAGE (μg/pg)		3.29 (1.78–5.18)	3.79 (1.77–7.59)	0.212

Data were presented as median (interquartile range) or count (percentage)^a^

Abbreviation: *ARB* angiotensin II receptor blocker; *ACEI* angiotensin-converting enzyme inhibitor, *HDL* high-density lipoprotein, *LDL* low-density lipoprotein, *ALT* alanine aminotransferase, *AST* aspartate aminotransferase, *eCCr* estimated creatinine clearance rate, *hs-CRP* high-sensitivity C-reactive protein, *ADMA* asymmetric dimethylarginine, *NT-proBNP* N-terminal pro-brain natriuretic peptide, *AGE* advanced glycation end product, *sRAGE* soluble form of receptor for AGE, *AGE/sRAGE* ratio of levels of AGE to soluble form of RAGE

### Association of AGE/sRAGE with albuminuria

The results of univariate and multivariate analysis were presented in Table 2. Variables with *P*-value < 0.1 in univariate analysis included SBP, DBP, pulse pressure, number of drugs, AST, N-terminal prohormone of brain natriuretic peptide (NT-proBNP), and AGE/sRAGE. Due to the collinearity among pulse pressure, systolic blood pressure and diastolic blood pressure, only pulse pressure was included into the multivariate analysis. The results of multivariate logistic regression models revealed that number of drugs (odds ratio [OR] = 1.986, 95% confident interval [CI] = 1.082–3.645, *P* = 0.027), NT-proBNP (OR = 1.015, 95% CI = 1.001–1.030, *P* = 0.030), and AGE/sRAGE (OR = 1.131, 95% CI = 1.001–1.278, *P* = 0.048) were independently associated with albuminuria (Table 2).

**Table 2:** Univariate and multivariate analysis for associated factors of albuminuria by logistic regression model with forward stepwise method

		Univariate		Multivariate	
		OR (95% CI)	*P*	OR (95% CI)	*P*
Age (year)		1.002 (0.97–1.035)	0.191		
Gender	Male	–	–		
	Female	0.936 (0.387–2.262)	0.883		
Smoking	Not smoker	–	–		
	Past smoker	0.915 (0.303–2.765)	0.875		
	Current smoker	0.677 (0.168–2.730)	0.584		
Body mass index		1.046 (0.909–1.202)	0.531		
Heart rate (/min)		1.001 (0.970–1.034)	0.928		
Systolic blood pressure (mmHg)		1.034 (1.010–1.059)	0.006		
Diastolic blood pressure (mmHg)		1.040 (1.006–1.076)	0.022		
Pulse pressure (mmHg)		1.040 (1.002–1.079)	0.038	1.020 (0.979–1.062)	0.350
Number of drugs		1.775 (1.074–2.934)	0.025	1.986 (1.082–3.645)	0.027
Impaired fasting glucose		1.206 (0.516–2.822)	0.665		
Laboratory					
Total cholesterol (mg/dl)		1.003 (0.991–1.016)	0.607		
Triglycerides (mg/dL)		1.001 (0.998–1.005)	0.541		
HDL (mg/dL)		1.002 (0.958–1.048)	0.927		
LDL (mg/dL)		1.001 (0.988–1.014)	0.933		
Fasting glucose (mg/dL)		1.002 (0.966–1.039)	0.931		
ALT (U/L)		0.979 (0.913–1.048)	0.538		
AST (U/L)		1.113 (0.998–1.241)	0.055	1.095 (0.962–1.246)	0.169
Uric acid (mg/dL)		1.220 (0.921–1.616)	0.166		
eCCr (mL/min)		0.989 (0.973–1.004)	0.149		
hs-CRP (mg/dL)		1.227 (0.501–3.002)	0.654		
Adiponectin (μg/mL)		0.994 (0.973–1.017)	0.622		
ADMA (μmol/L)		1.575 (0.003–732.211)	0.885		
NT-proBNP (pg/mL)		1.011 (1.000–1.022)	0.049	1.015 (1.001–1.030)	0.030
AGE (μg/mL)		1.208 (0.838–1.741)	0.311		
sRAGE (pg/ml)		0.999 (0.996–1.001)	0.189		
AGE/sRAGE (μg/pg)		1.109 (1.005–1.224)	0.039	1.131 (1.001–1.278)	0.048

Abbreviation: *ARB* angiotensin II receptor blocker, *ACEI* angiotensin-converting enzyme inhibitor, *HDL* high-density lipoprotein; *LDL* low-density lipoprotein; alanine aminotransferase, *eCCr* estimated creatinine clearance rate, *hs-CRP* high-sensitivity C-reactive protein, *ADMA* asymmetric dimethylarginine, *NT-proBNP* N-terminal pro-brain natriuretic peptide, *AGE* advanced glycation end product, *sRAGE* soluble form of receptor for AGE, *AGE/sRAGE* ratio of levels of AGE to soluble form of RAGE

## Discussion

To the best of our knowledge, this is the first study to investigate relationship of the ratio of AGE to sRAGE with micro- and macroalbuminuria in hypertensive patients. As our results showed a positive and independent association of AGE/sRAGE with albuminuria, the ratio may be a potentially surrogate biomarker for microvascular injury.

There is growing evidence that the AGE-RAGE axis plays a critical role in the development of atherosclerosis. In a cross-sectional study, Tahara et al. evaluated the correlation among adiponectin, AGE, sRAGE and AGE/sRAGE in 316 patients. The results showed that AGE/sRAGE was inversely related with adiponectin (β = − 0.126, *P* = 0.006), independent of the metabolic and clinical variables such as insulin resistance, triglycerides, HbA1c, HDL, age and sex. The significance correlation of AGE and sRAGE with adiponectin did not exist after adjusting for AGE/sRAGE in multiple regression analysis [19]. As adiponectin is an anti-inflammatory and vascular protective adipocytokine with insulin-sensitizing properties, the following study by Kajikawa et al. extended the observation by evaluating the association of AGE, sRAGE, and AGE/sRAGE with endothelial function measured by flow-mediated vasodilation. The results of multivariate regression analysis showed that AGE/sRAGE independently predicted flow-mediated vasodilation (β = − 0.23, *P* = 0.02) after adjusting several confounders, including age, sex, BMI, presence of hypertension, dyslipidemia, fasting glucose, and smoking status. Meanwhile, the association was not observed in either AGE or sRAGE alone [21]. Another study by Prasad et al. compared the AGE, sRAGE, endogenous secretory RAGE, cleaved RAGE between 88 patients with end-stage renal disease and 20 healthy people. Regarding the performance of identifying patients with end-stage renal disease, the results of receiver operating characteristic revealed that AGE/sRAGE had higher area under the curve of 0.918 than the ratio of AGE to endogenous secretory RAGE and the ratio of AGE to cleaved RAGE. The findings suggested that AGE/sRAGE is a better risk biomarker for end-stage renal disease [22]. Our study examined the role of AGE/sRAGE in relation to albuminuria, a strong clinical surrogate for cardiovascular diseases, in patients with essential hypertension. We observed that AGE/sRAGE was independently associated with presence of albuminuria (OR = 1.131, *P* = 0.048), but either AGE or sRAGE alone did not. Our results provide further support to the above evidence that AGE/sRAGE is a better biomarker for AGE-RAGE axis. Additionally, our results were also consistent with the above studies that AGE/sRAGE was positively associated with early-stage of atherosclerosis. However, the causal relationship between AGE/sRAGE and albuminuria as well as the prognostic performance of AGE/sRAGE deserve more investigations in the future longitudinal cohort study.

Many pharmaceutical studies have already applied AGE, RAGE, or sRAGE as a beneficial endpoint. Simvastatin stabilized plaque in type 2 diabetes by suppression of RAGE [24]. Enalapril/lercanidipine combination and nifedipine/telmisartan increased sRAGE [25, 26]. AGE were decreased after 6 and 12 months valsartan therapy [27]. But another study showed that irbesartan does not influence AGE in patients with type 2 diabetes and microalbuminuria [28]. Those findings triggered us to clarify the interactions between sRAGE and AGE and the clinical role of AGE/sRAGE as a biomarker, whereas the investigation of either AGE or sRAGE alone may result in insignificance [28]. In this way, we may understand more deeply the pharmaceutical benefits, which may decrease AGE formation and increase sRAGE effects to reduce cardiovascular risks [25, 26, 29–31]. Our study also found that the number of antihypertensive agents was also one of the associated factor of albuminuria. As the number of antihypertensive agents reflected the severity of hypertension and medication could influence the concentration of AGE and sRAGE, further studies are warranted to explore the causal relationship among AGE/sRAGE, medications, and clinical outcomes.

Some limitations should be considered in this study. First, the study population was composed of participants with hypertension merely; therefore, the results cannot be generalized to normal individuals. The results also may not applied in patients with previous or present cardiovascular events because this study did not include this group of patients. The observed association can only provide information for clinical judgment and risk stratification. Second, concentration of total sRAGE were measured; therefore, specific sRAGE variants could not be discriminated. Third, this is a cross-sectional study. Further studies will be necessary for the biomarker’s prognostic value.

## Conclusion

This study found that the ratio of AGE to sRAGE was independently associated with the existence of albuminuria in hypertensive patients. The results suggest that the ratio of AGE to sRAGE may be a surrogate biomarker for microvascular injury. Further prospective studies of the prognostic value of the ratio in relation to microvasular injury are needed.

## Abbreviations

AGE: Advanced glycation end-products
AST: Aspartate aminotransferase
BMI: Body mass index
DBP: Diastolic blood pressure
eCCr: Estimated creatinine clearance rate
ELISA: Enzyme-linked immunosorbent assay
HDL: High density lipoproteins
hsCRP: High-sensitive C-reactive protein
LDL: Low-density lipoprotein
NT-proBNP: N-terminal prohormone of brain natriuretic peptide
RAGE: Receptor for advanced glycation end-products
SBP: Systolic blood pressure
sRAGE: Soluble receptor for advanced glycation end-products
